# Characteristics of Women with Lung Adenocarcinoma in the World Trade Center Environmental Health Center

**DOI:** 10.3390/ijerph19137618

**Published:** 2022-06-22

**Authors:** Elaine Shum, Nedim Durmus, Sultan Pehlivan, Yuting Lu, Yian Zhang, Alan A. Arslan, Yongzhao Shao, Joan Reibman

**Affiliations:** 1 Division of Hematology and Medical Oncology, Department of Medicine, New York University Langone Health, New York, NY 10016, USA; elaine.shum@nyulangone.org; 2 Perlmutter Comprehensive Cancer Center, New York University, New York, NY 10016, USA; alan.arslan@nyulangone.org; 3 Division of Pulmonary Medicine, Department of Medicine, New York University Langone Health, New York, NY 10016, USA; nedim.durmus@nyulangone.org (N.D.); sultan.pehlivan@nyulangone.org (S.P.); 4 World Trade Center Environmental Health Center, NYC Health + Hospitals, New York, NY 10016, USA; yuting.lu@nyulangone.org (Y.L.); yian.zhang@nyulangone.org (Y.Z.); yongzhao.shao@nyulangone.org (Y.S.); 5 Division of Biostatistics, Department of Population Health, New York University Langone Health, New York, NY 10016, USA; 6 Department of Obstetrics and Gynecology, New York University Langone Health, New York, NY 10016, USA; 7 Division of Epidemiology, Department of Population Health, New York University Langone Health, New York, NY 10016, USA

**Keywords:** World Trade Center (WTC), WTC Environmental Health Center, September 11, lung cancer, lung adenocarcinoma, women, smoking, biomarker

## Abstract

The destruction of the World Trade Center towers on 11 September 2001 exposed local residents, workers, and individuals in the area (Survivors) to dust and fumes that included known and suspected carcinogens. Given the potential for inhalation of toxic substances and the long latency after exposure, the incidence of lung cancer is expected to increase in WTC-exposed individuals. We describe the characteristics of women WTC Survivors with lung adenocarcinoma who were enrolled in the WTC Environmental Health Center (WTC EHC) between May 2002 and July 2021. A total of 173 women in WTC EHC had a diagnosis of any type of lung cancer, representing 10% of all cancers in women. Most of the lung cancers (87%) were non-small cell carcinomas, with adenocarcinoma (77%) being the most common subtype. Nearly half (46%) of these patients were exposed to dust clouds on 11 September 2001. Race and ethnicity varied by smoking status, as follows: 44% of Asian women compared with 29% of non-Hispanic White women were never-smokers (*p* < 0.001). There was no significant difference between the pathologic characteristics of adenocarcinomas between never and ever smokers. We also summarize EGFR, ALK, KRAS, ROS-1 and BRAF mutation status stratified by smoking, race and ethnicity. The identification of a relatively high proportion of women never-smokers with lung cancer warrants further investigation into the role of WTC dust exposure.

## 1. Introduction

The environmental disaster created by the destruction of the World Trade Center (WTC) towers and surrounding buildings on 11 September 2001 affected large groups of community members (WTC Survivors) as well as rescue workers and responders. WTC Survivors include local residents, local workers, children, students and commuters. Many had acute exposure to the WTC dust from the collapse of the buildings, which released approximately 106 tons of material and/or chronic exposure from resuspended dust and fires that continued through December 2001 [1,2,3,4,5]. Components of the dust and fumes include respirable particulate matter (PM10 and PM2.5) containing a combination of pulverized cement, glass fibers, asbestos, lead and combustion products, as well as complex mixtures of volatile and potentially carcinogenic chemicals including polycyclic aromatic hydrocarbons, polychlorinated biphenyls and polychlorinated furans and dioxins [1,6,7].

Lung cancer is the leading cause of cancer death in both men and women in the United States, accounting for approximately 22% of cancer deaths with a 5-year survival rate of 21.7% between 2011 and 2017 [8]. Cigarette smoking is by far the most important risk factor for lung cancer [9,10,11,12]. Although lung cancer death rates declined by 19% from 2002 to 2015 among women due to reduced tobacco use, the proportion of lung cancer in never-smokers has been increasing, raising the question of other environmental exposures as risks [13,14]. Lung cancer in never-smokers is most commonly adenocarcinoma, which may be molecularly distinct from smoking-associated cancers with an increased incidence of specific driver mutations. For example, EGFR mutations are the most common oncogenic driver in East Asian populations, with an incidence of approximately 40% [15,16,17]. In general, our understanding of the genomic landscape of lung cancer in nonsmokers is not as well characterized as that in smokers; most genomic studies in lung cancer have been determined from samples derived from smokers. Moreover, although tobacco use is a well-described cause of lung cancer, the contribution of environmental or occupational exposures to lung cancers and their effect on genomic modifications remains incompletely described [18,19,20,21,22,23].

The WTC Environmental Health Center (WTC EHC) is a surveillance and treatment program for WTC Survivors under the Centers for Disease Control/National Institute of Occupational and Environmental Health (CDC/NIOSH). Many of these patients enrolled with a diagnosis of cancer are considered a certifiable condition under the H.R. 847 James Zadroga Health and Compensation Act of 2010 (Zadroga Act) [24,25]. In contrast to the WTC Responder programs, nearly 50% of the patients in the WTC EHC are women [5] and lung cancer is the second most common cancer in these women [24]. The data collected related to WTC exposure provides an opportunity to investigate the contribution of environmental exposures to the clinical and genomic characteristics of these lung cancers. In this report, we provide characteristics of the lung cancer cases in women WTC Survivors in the WTC EHC, with a focus on adenocarcinoma cases identified as of 1 July 2021.

## 2. Materials and Methods

### 2.1. Patient Enrollment in the WTC EHC

The WTC EHC was created in response to community requests in the years after 11 September 2001 and was included as the Center of Excellence for WTC Survivors in the CDC/NIOSH WTC Health Program (WTCHP) under the Zadroga Act [26]. Patients self-refer to this program and, under law, enrollment requires the presence of a defined WTC exposure and a certifiable WTC-related health condition, which includes specific cancers identified within a defined time period, such as lung cancer. Community members (Survivors) must document their location and activities on 11 September 2001, as well as time periods and hours spent in the 1.5 m radius of the former WTC complex in the days and weeks following the disaster. These activities might include living and/or working in the area. The participants of WTC EHC have to have certifiable health conditions related to WTC exposure according to the law. Federal government rules include specific geographic boundaries (roughly south of Houston Street and some western areas of Brooklyn) and time periods (from 11 September 2001 to 31 July 2002) during which a community member can be considered as exposed. The possibility exists that additional exposures might contribute to the risk of developing cancer. Since tobacco smoke is a major risk for lung cancer, we also include rates of tobacco use in our analysis and discussion. Patients in the WTC EHC undergo standardized medical and mental health evaluations at baseline and follow-up monitoring visits.

All subjects in the WTC EHC were asked to provide informed consent to participate in research and only those who signed consent were included in this analysis. The study was conducted in accordance with the Declaration of Helsinki, and the protocol was approved by the New York University School of Medicine Institutional Review Board (IRB number: i06-1). Patients with cancer were analyzed after removal of personal identifiers with IRB approval to review de-identified data (IRB number: i06-1_MOD49). Documentation of consent to be re-contacted is included for subsequent studies.

### 2.2. Cancer Information

Cancer information for patients in this study was obtained from the WTC EHC database with the WTC EHC Pan-Cancer Database (WTC EHC PCDB) and the New York State Cancer Registry (NYSCR). Patients can self-refer into the WTC EHC with a previous diagnosis of cancer, or a cancer diagnosis may be made subsequent to enrollment in the WTC EHC. In addition, we reviewed data from linkage with the NYSCR with data available as of 1 August 2019 and imported data to WTWC EHC PCDB. Data from the WTC EHC PCDB also interfaces with the WTC EHC clinical databases [25]. Lung cancer diagnoses as of 1 July 2021 at WTC EHC were verified from pathology reports and data was extracted from pathology reports, clinical records and other available medical records. Lung cancer characteristics such as age at diagnosis, anatomic location of tumor, ICD-10 classification, tumor size, grade, histology (ICD-O-3 code), TNM (Tumor size, Node involvement, Metastases status) classification, cancer stage and available cancer biomarker information were recorded for each case of lung adenocarcinoma. Information on cancer biomarkers was obtained from pathology reports and medical records including physician progress notes with a focus on the common biomarkers for lung cancer (EGFR, ALK, KRAS, ROS-1 and BRAF mutation).

### 2.3. Exposure Information

Information about environmental exposures including WTC dust or fume exposures and tobacco use was obtained from questionnaires administered to each enrollee at the initial visit to WTC EHC and subsequent monitoring visits. Community members were potentially exposed to massive amounts of dust on 11 September 2001 (acute exposure, dust cloud yes/no). In addition, WTC Survivors may have had chronic exposure to resuspended dust or from the fires that burned through December 2001. For our initial analysis, we simplified categories of exposure as those with “acute” exposure, e.g., they were exposed to the dust clouds on 11 September 2001 (dust cloud: yes). We then included potential for chronic exposure, which depended on the category of activity, i.e., local resident, local worker, student, clean-up worker. These categories are not mutually exclusive as participants may have had both dust cloud exposure on 11 September 2001 and the potential for chronic exposure as a local resident, local worker, or clean-up worker. The WTC EHC questionnaires also collect information on basic exposures including occupational and lifestyle exposures (e.g., smoking). Never-smokers were defined as those reporting a ≤ 1 pack-year (p-y) and ever-smokers as those reporting >1 p-y history of tobacco use. Tobacco history was not available for a few lung adenocarcinoma cases (*n* = 11).

### 2.4. Data Analysis

Descriptive statistics were used to summarize WTC exposure and demographic characteristics of women lung cancer patients including median and range for continuous variables and counts and percentages for binary or categorical variables. Tumor characteristics were compared by smoking history using chi-square tests and Fisher’s exact test for categorical variables and Mann–Whitney test for continuous variables. Distribution of age of diagnosis for every 5-year period stratified by smoking history was summarized using bar graphs. Biomarkers statuses are summarized and tabulated based on what is available and *p*-values of the Fisher exact test are calculated within the subgroup of patients with available biomarker data for each of the biomarkers. Statistical analyses were performed using R software (version 3.6.3) (R Core Team, Vienna, Austria).

## 3. Results

### 3.1. Participants

**Figure 1:**
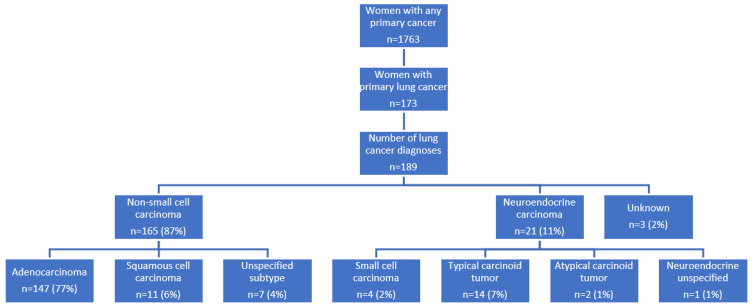
Frequency of women lung cancers and histological distribution at the WTC EHC as of 1 July 2021.

Among patients enrolled in the WTC EHC between May 2002 and 1 July 2021, 3759 had a cancer diagnosis; 47% (*n* = 1763) were women. Primary lung cancer was identified in 305 patients; 57% (*n* = 173) were women. Primary lung cancer accounted for 10% of all female cancers in the WTC EHC population. Sixteen women had more than one primary lung cancer diagnosis, resulting in a total of 189 total lung cancer diagnoses in women WTC Survivors. The histologic subtypes of the cancer diagnoses are shown (Figure 1). Of the lung cancer diagnoses, 165 (87%) were any type of non-small cell carcinoma, 21 (11%) any type of neuroendocrine carcinoma and 3 (2%) of unknown histology. More specifically, among the 189 lung cancer diagnoses, 147 (77%) were adenocarcinoma, 11 (6%) squamous cell carcinoma and 7 (4%) unspecified subtype diagnoses. Twenty-one patients with neuroendocrine carcinoma included 4 patients (2%) with small cell carcinoma, 14 (7%) with a typical carcinoid tumor, 2 (1%) with atypical carcinoid tumors and 1 (1%) with an unspecified subtype diagnosis. Three patients (2%) had an unknown histological type. (Figure 1).

Since the predominant histologic type of cancer was adenocarcinoma (77%), we focused on basic exposure and demographic characteristics of women with lung adenocarcinoma stratified by available smoking history (Table 1). Among patients with lung adenocarcinoma, 136 patients had an available smoking history with a similar distribution of never and ever-smokers at 49% (*n* = 67) and 51% (*n* = 69), respectively. The median age on 9/11/01 was 50 years (range 27–69) with no statistically significant difference between never- and ever-smokers. The median age of diagnosis was 63 (range 34–85) with no statistically significant difference between never (62 years) and ever-smokers (64 years). The median latency period for diagnosis from 11 September 2001 was 13.9 years (range 3–19) with no statistically significant difference between never (13.8 years) and ever-smokers (14.5 years) (Table 1).

Women with lung adenocarcinoma had a diverse race and ethnicity composition, with 52% White, 28% Asian, 16% Black or African American and 4% Hispanic. The distribution of race and ethnicity varied by smoking status with 56% of never-smokers identified as Asian and only 3% of ever-smokers identified as Asian. In contrast, only 29% of never-smokers identified as White, while 71% of ever-smokers identified as White (*p* < 0.001) (Table 1).

Among women lung adenocarcinoma patients, 46% were exposed to the dust cloud on 11 September 2001, with a similar distribution among never and ever-smokers (46%). Most never-smokers were residents (58%) and the majority of the ever-smokers were local workers (63%) (Table 1).

Distribution of the age at diagnosis of lung adenocarcinomas is shown in Figure S1.

### 3.2. Pathologic Characteristics

Pathologic characteristics of adenocarcinoma in women in the WTC EHC are shown in Table 2. No statistically significant difference was found between never-smokers and ever-smokers in a grade of differentiation, although more never-smokers were grade 3 (25%) than grade 2 (21%) and more ever-smokers were grade 2 (29%) than grade 3 (17%). Unfortunately, nearly 42% of never-smokers and 38% of ever-smokers were with unknown grade (Table 2). Using the TNM staging system, most of the never-smokers (49%) and ever-smokers (51%) were classified as T1 without any regional lymph node metastases (N0) in 45% of never-smokers and 58% of ever-smokers. Nineteen percent of the never-smokers and 20% of ever-smokers had any distant metastasis at the time of diagnosis (Table S1). Using the TNM staging system, at the time of diagnosis, 39% of never-smokers and 46% of ever-smokers were diagnosed with Stage 1 with no statistically significant difference (Table 2).

### 3.3. Biomarker Status in Women Lung Adenocarcinomas

Available biomarker status for EGFR, ALK, KRAS, ROS-1 and BRAF is summarized in Table 3 according to smoking history. EGFR mutations were found in 85% of never-smokers (*n* = 33), whereas the majority (78%) were negative ever-smokers (*n* = 36). For the 35 patients where KRAS mutational testing was performed, 83% were negative in 12 never-smokers and 78% were positive in 23 ever-smokers. Although we have small numbers of EGFR and KRAS mutations among available mutation results, there appeared to be a statistically significant difference between never and ever-smokers for both of these biomarkers based on the simple chi-square test. For ROS-1 mutation status, only one patient had a positive result in never-smokers and all patients in ever-smokers were negative. Only one patient was noted as positive in an ever-smoker for the BRAF mutation (Table 3).

The subtypes of EGFR and KRAS mutations are summarized in Table S2. There was no statistically significant difference between those subtypes of EGFR and KRAS separated by smoking history.

The biomarker status was further stratified by race and ethnicity. EGFR was positive in 31% of 32 NH-White women and all Asian women were positive for an EGFR mutation (*n* = 18 mutations). The one positive (10%) among ten ALK mutations was noted in an Asian patient. KRAS was predominantly positive in 92% of NH-White women among the available 13 patients tested for KRAS mutations (Table 4).

## 4. Discussion

The characteristics of lung cancers in the WTC EHC were previously described by our group, demonstrating that more than half of all lung cancers occurred in women, and of these, a high proportion were noted in never-smokers (48%) [27]. This is particularly important given the reported 15% incidence of lung cancer in women never-smokers in the U.S. [28]. This study further explores the risk of lung cancer in those exposed to the WTC disaster, where the risks of cancer development remain incompletely understood. As the most common histologic subtype of lung cancer in never-smokers is adenocarcinoma, we focused on adenocarcinomas in women enrolled in the WTC EHC program.

Among women with a diagnosis of lung adenocarcinoma, 49% were never-smokers. The majority of adenocarcinoma cases were non-Hispanic White (52%), followed by Asian (28%). One possibility for this distribution may be the location of the WTC disaster area, which encompasses the New York Chinatown area. The proportion of adenocarcinomas among never-smokers was the highest in Asian women (56%), in accordance with the historically increased incidence of lung cancer in Asian never-smoker women.

Lung cancers in never-smokers are more likely to have driver mutations [28,29,30,31], but otherwise do not have well-established risk factors, adding to the complexity of understanding this subset. We retrospectively analyzed this cohort for the presence of the most common clinically assessed molecular alterations, EGFR, ALK, ROS1, KRAS and BRAF. The recent drug approval for sotorasib for metastatic lung cancer with KRAS G12C mutations has increased interest in the presence of this mutation [32]. EGFR testing was conducted and available to us for 69 patients, of which 85% of never-smokers were noted to be EGFR positive. EGFR mutations are reported in approximately 15% of cases, although up to 40% in Asian populations [15,16,17,33]. All of the available EGFR test results that were identified (*n* = 18) in Asian women were positive. KRAS mutations are more commonly associated with lung cancers diagnosed in smokers and are reported to be positive in about 40% of cases [34,35]. We identified 78% of patients to have KRAS mutations in those tested in our population.

We identified a high proportion of women never-smokers in this lung cancer cohort from the WTC EHC compared to lung cancer patients in the general population [8]. One reason for this high rate of never-smokers may be the high proportion of Asian women in our population. This population represents a unique subset of patients both for understanding the role of WTC exposures in lung cancer development as well as studying lung cancer in non-smoker women as a whole. Further molecular and mutational signature analysis of the tumors of these women may identify distinct genetic signatures. Recently, Jasra and colleagues [36] exposed mice to WTC particulate matter and the hematopoietic stem cells were collected, revealing murine mutational signatures closely related to COSMIC signatures associated with tobacco smoke. They also noted an increased burden of clonal hematopoiesis in first responders with WTC exposure compared to non-WTC exposed firefighters suggesting a link between WTC dust exposure with increased genotoxic stress and inflammation [36].

The high proportion of women never-smokers with WTC exposure suggests that this is a potentially high-risk population for lung cancer and raises the question of the need for increased surveillance. Currently, lung cancer screening by low-dose CT scans of the chest is limited to former or current heavy smokers in the general population; however, the increased incidence of lung cancer in women never-smokers in the WTC EHC, as well as the respiratory aspect of WTC dust exposure, supports the rationale for considering screening in this population of women. Importantly, the majority (38%) of never-smokers in this cohort were diagnosed with stage 1 lung cancer, whereas lung cancer in never-smokers is more commonly diagnosed in later stages. Reasons for these early diagnoses are currently unknown, but provider bias or heightened awareness of the possibility of malignancy in a WTC dust-exposed patient may contribute to this early detection, again reinforcing the need to explore lung cancer screening in this group.

This study has important strengths. In contrast to the Responders, the WTC EHC includes nearly 50% women and is diverse in race and ethnicity. This diversity is reflected in the patients with lung cancer, among whom more than 50% were women. The WTC EHC PCDB includes the information to identify patients who have agreed to be recontacted and have documentation of the location of diagnosis and biopsy specimens [25]. The collection of biomarker data adds to the strengths of the study. In addition, the continued surveillance and follow-up of this population allows for an improved understanding of the behavior of lung cancer in women over time. Subsequent analyses may allow further understanding of the interaction between tobacco use and WTC exposure.

There are several limitations to this study. Many patients in the WTC EHC self-refer when they have been diagnosed with cancer, and so the determination of the incidence of cancer in the WTC EHC population is limited. As a result, we are unable to distinguish whether lung cancers were detected because of increased monitoring and screening of this population. Early-stage lung cancer detection in our population may be due to the use of advanced diagnostic imaging or regular medical screening leading to early cancer detection. Many of the lung cancers were self-referred and thus not identified through the screening in the WTC EHC. As such, we cannot report incidence or prevalence of lung cancer in this population; these studies can be provided by the NYC Department of Health WTC Health Registry, with an ongoing defined cohort. Thus, meaningful direct comparisons cannot be made between our population and the general population. Furthermore, the enrollment time after 11 September varied over more than 10 years as the WTC Survivor cohort is still open to enrollment for qualified patients to date. Association analyses on cancer-exposure relations without suitable adjustments for enrollment dates are potentially biased. Organizing and standardizing the very complex WTC-related exposure data as well as occupational and other exposure data for future cancer-exposure association studies is necessary. While our retrospective biomarker analysis was fairly comprehensive, it is limited due to several factors, including that many lung cancers were diagnosed before molecular testing for biomarkers, such as EGFR, ALK, ROS1 and others, were considered standard of care. For context, the discovery of the EGFR mutation in lung cancer was in 2004 [37]. Recent years have made next-generation sequencing (NGS) of tumors more widespread and used in clinical practice. In addition, a large proportion of the lung cancers were diagnosed at stage 1, when NGS testing is not universally covered by health insurance and so may not have been requested or conducted. It should be pointed out that the limitation of the biomarker data tabulated here being available only on a part of the study subjects is that any formal data analysis can have potential biases, including the bias due to data missing not-at-random. More systematic collection and analysis of these biomarkers in the future are warranted. The flexibility of the WTC EHC PCDB allows for the inclusion of future biomarkers, and further research studies are being planned to potentially perform biomarker analyses on archival tissue.

## 5. Conclusions

We provide a description of lung adenocarcinoma in women in the Survivor population with documented exposure to the WTC dust and fumes. As it is now twenty years since 11 September 2001, we expect that more lung cancers will be identified in the future given the latency period of lung cancer development. The identification of a relatively high proportion of women never-smokers with lung cancer in this population warrants further investigation into the role of WTC dust exposure and whether this exposure may contribute to the generation of a unique mutational signature. In addition, the potential role of lung cancer screening in women non-smoker Survivors deserves further attention. This study sets the stage for future studies centered not only on lung cancer in women but also in nonsmokers, given the potential influence of WTC dust exposure as a causative environmental exposure.

## Figures and Tables

**Table 1:** Characteristics of women lung adenocarcinoma patients in the World Trade Center Environmental Health Center (WTC EHC) with available smoking history.

	Level	Overall	Never (≤1 p-y)	Ever (>1 p-y)	*p*
*n*		136	67	69	
Age at 9/11 (Median [range])		49.7 [27.5, 69.4]	47.4 [27.5, 69.4]	50.7 [27.7, 63.2]	0.838
Age at diagnosis (Median [range])		63 [34, 85]	62 [38, 85]	64 [34, 82]	0.588
Latency period by year (Median [range])		13.9 [3.3, 19.7]	13.8 [4.7, 18.4]	14.5 [3.3, 19.7]	0.365
Race/Ethnicity (%)	NH-White	61 (51.7)	16 (29.1)	45 (71.4)	<0.001
	Asian	33 (28.0)	31 (56.4)	2 (3.2)	
	NH-Black	19 (16.1)	7 (12.7)	12 (19.0)	
	Hispanic	5 (4.2)	1 (1.8)	4 (6.3)	
	Total	136 (100)	55 (100)	63 (100)	
Caught in WTC cloud (%)	No	64 (54.2)	30 (54.5)	34 (54.0)	1
	Yes	54 (45.7)	25 (45.5)	29 (46.0)	
	Total	110 (100)	55 (100)	63 (100)	
Exposure category (%)	Worker	56 (47.9)	17 (30.9)	39 (62.9)	0.001
	Resident	49 (41.9)	32 (58.2)	17 (27.4)	
	Total	105 (100)	49 (100)	56 (100)	

**Table 2:** Characteristics of women lung adenocarcinomas in the WTC Environmental Health Center with smoking history.

	Level	Overall	Never (≤1 p-y)	Ever (>1 p-y)	*p*
*n*		136	67	69	
Grade (%)	G1. Well-differentiated	19 (14.0)	8 (12.0)	11 (16.0)	0.402
	G2. Moderately differentiated	34 (25.0)	14 (20.9)	20 (29.0)	
	G3. Poorly differentiated	29 (21.3)	17 (25.3)	12 (17.4)	
	G4. Undifferentiated	0 (0.0)	0 (0.0)	0 (0.0)	
	Unknown	54 (39.7)	28 (41.8)	26 (37.6)	
	Total	136 (100)	67 (100)	69 (100)	
Stage (%)	0	7 (5.1)	5 (7.5)	2 (2.9)	0.639
	1	58 (42.6)	26 (38.8)	32 (46.4)	
	2	13 (9.6)	5 (7.5)	8 (11.6)	
	3	18 (13.2)	10 (14.9)	8 (11.6)	
	4	27 (19.9)	13 (19.4)	14 (20.3)	
	Unknown	13 (9.6)	8 (11.9)	5 (7.2)	
	Total	136 (100)	67 (100)	69 (100)	

**Table 3:** Biomarker status of women lung adenocarcinomas in the WTC Environmental Health Center with smoking history.

		Never (≤ 1 p-y)		Ever (>1 p-y)		
Biomarker	Total Available (*n* *)	Negative (%)	Positive (%)	Negative (%)	Positive (%)	*p* **
EGFR	69	5 (15)	28 (85)	28 (78)	8 (22)	<0.001
ALK	49	21 (95)	1 (5)	27 (100)	0	0.449
KRAS	35	10 (83)	2 (17)	5 (22)	18 (78)	0.001
ROS-1	15	4 (75)	1(25)	10 (100)	0	NA
BRAF	5	0	0	4 (75)	1 (25)	1

(*n* * denotes the total number of patients tested for the specified available biomarker; percentages are calculated for each smoking group; *p* ** denote the *p*-value of the Fisher exact test based on patients with available biomarker information.).

**Table 4:** Biomarker status of women lung adenocarcinomas in the WTC Environmental Health Center separated by race and ethnicity.

		Hispanic		NH-White		NH-Black		Asian	
Biomarker	Total Available (*n* *)	Negative (%)	Positive (%)	Negative (%)	Positive (%)	Negative (%)	Positive (%)	Negative (%)	Positive (%)
EGFR	62	2 (50)	2 (50)	22 (69)	10 (31)	6 (75)	2 (25)	0	18 (100)
ALK	44	1 (100)	0	25 (100)	0	8 (100)	0	9 (90)	1 (10)
KRAS	31	3 (75)	1 (25)	1 (8)	12 (92)	2 (29)	5 (71)	6 (86)	1 (14)
ROS-1	13	0	0	8 (100)	0	3 (75)	1(25)	1 (100)	0
BRAF	4	0	0	3 (100)	0	0	1 (100)	0	0

(*n* * denotes the total number of patients tested for the specified available biomarkers; percentages are calculated for each race/ethnicity group).

## Data Availability

The datasets are not publicly available, but deidentified and anonymized information is potentially available upon reasonable request.

## Supplementary Materials

The following supporting information can be downloaded at: https://www.mdpi.com/article/10.3390/ijerph19137618/s1, Table S1. TNM classification of women lung adenocarcinomas in the WTC Environmental Health Center with smoking history; Table S2. Subtypes of EGFR and KRAS mutations status of women lung adenocarcinomas in the WTC Environmental Health Center with smoking history (KRAS Codon 12 unspecified subtype includes G12B, G12D, G12F and G12V subtypes). Figure S1. Distribution of age of diagnosis in women lung adenocarcinoma patients in the WTC EHC. Smoking data is not available for 11 patients.
