# Supplementary material for: Characteristics of Women with Lung Adenocarcinoma in the World Trade Center Environmental Health Center
Source: Int J Environ Res Public Health. 2022 Jun 22;19(13):7618. doi: 10.3390/ijerph19137618 (PMC9265949; doi:10.3390/ijerph19137618)
Supplement: Supplementary file 1 [file ijerph-19-07618-s001.zip › ijerph-1728364-supplementary.pdf]

**Table S1.** TNM classification of women lung adenocarcinomas in the WTC Environmental Health Center with smoking history

| | Level | Overall | Never (<=1 p-y) | Ever (>1 p-y) | *p* |
|---|---|---|---|---|---|
| *n* | | 136 | 67 | 69 | |
| **pT (Primary tumor) (%)** | T1 | 68 (50.0) | 33 (49.3) | 35 (50.7) | 0.675 |
| | T2 | 20 (14.7) | 7 (10.4) | 13 (18.8) | |
| | T3 | 5 (3.7) | 2 (3.0) | 3 (4.3) | |
| | T4 | 5 (3.7) | 3 (4.5) | 2 (2.9) | |
| | Tis | 7 (5.1) | 5 (7.5) | 2 (2.9) | |
| | Unknown | 31 (22.8) | 17 (25.3) | 14 (20.3) | |
| **pN (Regional lymph nodes) (%)** | N0. No regional lymph node metastasis | 70 (51.5) | 30 (44.8) | 40 (58.0) | 0.511 |
| | N1. Regional lymph node metastasis | 32 (23.5) | 18 (26.9) | 14 (20.3) | |
| | Unknown | 34 (25.0) | 19 (28.3) | 15 (21.7) | |
| **pM (Distant metastasis) (%)** | M0. No distant metastasis | 96 (70.6) | 46 (68.7) | 50 (72.5) | 0.675 |
| | M1. Distant metastasis | 27 (19.8) | 13 (19.4) | 14 (20.3) | |
| | Unknown | 13 (9.6) | 8 (11.9) | 5 (7.2) | |

**Table S2.** Subtypes of EGFR and KRAS mutations identified in women lung adenocarcinomas in the WTC Environmental Health Center with smoking history (KRAS Codon 12 unspecified subtype includes G12B, G12D, G12F and G12V subtypes).

| | | | | Never (<=1 p-y) | | Ever (>1 p-y) | | |
|---|---|---|---|---|---|---|---|---|
| **Biomarker** | **Overall (n)** | **Subtype** | **Total** | **Negative (n)** | **Positive (n)** | **Negative (n)** | **Positive (n)** | ***p*** |
| **EGFR** | 88 | Exon 18 mutation | 3 | 1 | 0 | 2 | 0 | NA |
| | | Exon 19 deletion mutation | 23 | 2 | 14 | 5 | 2 | 0.011 |
| | | Exon 20 insertion mutation | 9 | 3 | 3 | 2 | 1 | 1 |
| | | Exon 21 mutation | 18 | 3 | 8 | 3 | 4 | 0.627 |
| | | Unspecified subtype | 35 | 4 | 5 | 24 | 2 | 0.006 |
| **KRAS** | 39 | Codon G12C | 5 | 0 | 0 | 0 | 5 | NA |
| | | Codon 13 | 1 | 1 | 0 | 0 | 0 | NA |
| | | Codon 12 unspecified | 7 | 1 | 1 | 0 | 5 | 1 |

| | | | | | | |
|---|---|---|---|---|---|---|
| | Unspecified subtype | 23 | 9 | 1 | 5 | 8 | 0.029 |

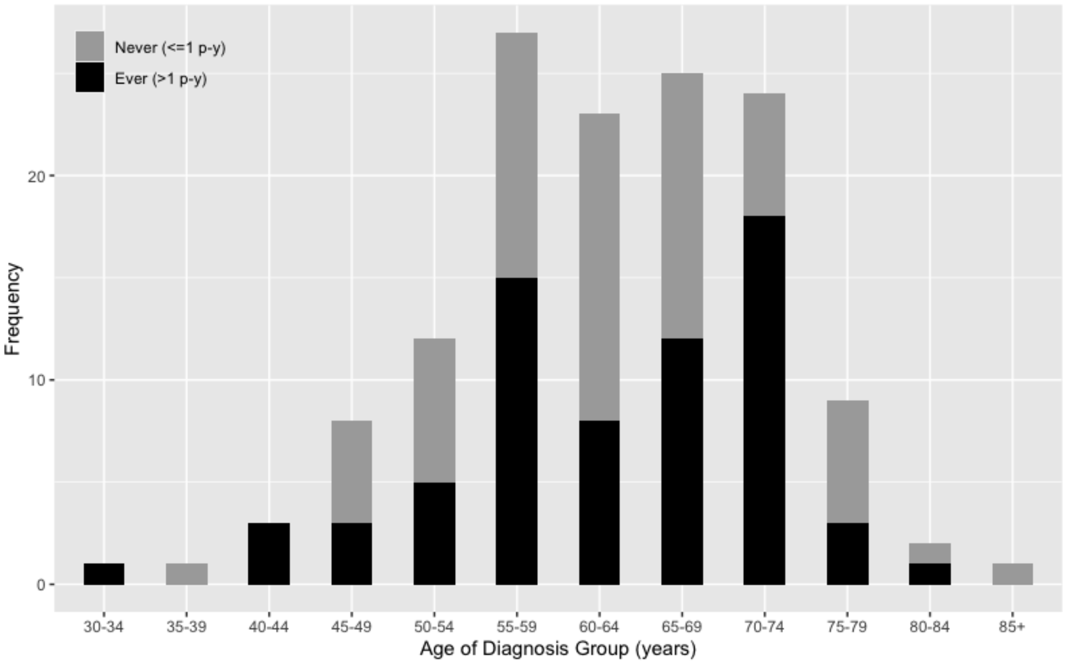


**Figure S1.** Distribution of age of diagnosis in women lung adenocarcinoma patients in the WTC EHC. Smoking data is not available for 11 patients.
